# Long-Term Functional Outcome of Surgical Treatment for Thoracic Outlet Syndrome

**DOI:** 10.3390/diagnostics8010007

**Published:** 2018-01-12

**Authors:** Jesse Peek, Cornelis G. Vos, Çağdas Ünlü, Michiel A. Schreve, Rob H. W. van de Mortel, Jean-Paul P. M. de Vries

**Affiliations:** 1 Department of Vascular Surgery, St Antonius Hospital, 3435CM Nieuwegein, The Netherlands; j.peek@students.uu.nl (J.P.); r.van.de.mortel@antoniusziekenhuis.nl (R.H.W.v.d.M.); 2 Department of Vascular Surgery, Medical Center Alkmaar, 1815JD Alkmaar, The Netherlands; ncgvos@gmail.com (C.G.V.); cagdas.unlu@nwz.nl (Ç.Ü.); m.schreve@nwz.nl (M.A.S.)

**Keywords:** thoracic outlet syndrome, first rib resection, surgical procedures, operative, patient reported outcome measures

## Abstract

First rib resection for thoracic outlet syndrome (TOS) is clinically successful and safe in most patients. However, long-term functional outcomes are still insufficiently known. Long-term functional outcome was assessed using a validated questionnaire. A multicenter retrospective cohort study including all patients who underwent operations for TOS from January 2005 until December 2016. Clinical records were reviewed and the long-term functional outcome was assessed by the 11-item version of the Disability of the Arm, Shoulder, and Hand (QuickDASH) questionnaire. Sixty-two cases of TOS in 56 patients were analyzed: 36 neurogenic TOS, 13 arterial TOS, 7 venous TOS, and 6 combined TOS. There was no 30-day mortality. One reoperation because of bleeding was performed and five patients developed a pneumothorax. Survey response was 73% (*n* = 41) with a follow-up ranging from 1 to 11 years. Complete relief of symptoms was reported postoperatively in 27 patients (54%), symptoms improved in 90%, and the mean QuickDASH score was 22 (range, 0–86). Long-term functional outcome of surgical treatment of TOS was satisfactory, and surgery was beneficial in 90% of patients, with a low risk of severe morbidity. However, the mean QuickDASH scores remain higher compared with the general population, suggesting some sustained functional impairment despite clinical improvement of symptoms.

## 1. Introduction

Thoracic outlet syndrome (TOS) is caused by compression of the neurovascular bundle (brachial plexus, subclavian vein or artery) in the thoracic outlet. TOS can be grouped as neurogenic and vascular, depending on the anatomical structure that is compromised. Neurogenic TOS (NTOS), the most common form (95–99%), is caused by compression of the brachial plexus. Neurological symptoms, such as pain, paresthesia, numbness, Raynaud phenomenon, and/or weakness in arm and shoulder, have been described. Vascular TOS, caused by compression of the subclavian vessels below the clavicle, includes venous TOS (VTOS) and arterial TOS (ATOS) and is relatively uncommon [1,2].

TOS, especially NTOS, is a poorly understood condition, and the diagnosis is highly debatable because there are no objective, well-defined, diagnostic criteria. Furthermore, diagnosing NTOS is a challenging task because of the variability of presenting symptoms and the lack of sensitive and specific diagnostic tests.

Physiotherapy and additional medication (i.e., painkillers, anti-inflammatory medications, or muscle relaxants) are the mainstays of management of NTOS, and together they may improve arm function and reduce symptoms. If conservative treatment fails, surgical treatment may be considered for patients with persisting symptoms [2,3]. Management and presentation are different in the case of vascular involvement, and surgery is preferred in a larger proportion of patients [2,3].

Many publications have appeared in recent decades documenting different surgical approaches and their outcomes. Surgical interventions seem beneficial for most patients, although patient selection is important [4]. Significant improvements in arm function in both the neurogenic and vascular group was observed [4]. However, high-quality studies, including those with large enough sample sizes and using validated outcome measures to describe results of treatment, are lacking. Thus, the main objective of this study was to evaluate the long-term functional outcomes for surgically treated patients with TOS.

## 2. Materials and Methods 

The Medical Ethics Review Boards of St. Antonius Hospital (MEC-U Nieuwegein) and Medical Center Alkmaar (METC Noord-Holland, Alkmaar, The Netherlands) The Netherlands approved the study (6 July 2016, R&D/Z16.061), and informed consent was obtained for all patients.

### 2.1. Patient Selection

This retrospective, multicenter, medical record review was performed at the departments of vascular surgery of two large vascular referral centers. The study enrolled all patients with unilateral or bilateral first or cervical rib resection for TOS of any type from January 2005 until December 2016. The extracted data included patient characteristics, type of TOS, and the presenting symptoms and signs, including pain, numbness, and loss of strength in arm, neck, or shoulder. Thrombotic signs, medical history, preceding trauma, risk factors, intoxications, and other complaints were also recorded. Information from the diagnostic workup included data of physical examination (e.g., Roos Elevated Arm Stress Test, Adson test, and Allen test) and data from additional radiographic imaging, including phlebographies, arteriographies, duplex ultrasonography with additional provocation testing, computed tomography scans (CT), or magnetic resonance imaging (MRI). Also collected was the type of operation, outcomes of additional therapy (thrombolysis, percutaneous transluminal angioplasty, or vascular reconstruction), and follow-up information.

### 2.2. Workup and Surgical Intervention

The workup for patients with symptoms indicating TOS depended on the type of TOS. An important part of the work-up for NTOS patients consisted of ruling out other pathology that could cause similar symptoms. This included imaging studies—such as duplex ultrasonography with provocation testing, CT, or MRI—and referral to a neurologist to rule out other neurogenic causes such as radicular compression or peripheral nerve compression. The first step in treatment was always a conservative management, including counseling, physiotherapy and injections with anesthetics, steroids, or Botox. When conservative management failed to improve symptoms, surgery for thoracic outlet decompression was offered.

VTOS patients, usually presenting with venous thrombosis, were treated conservatively (i.e., anticoagulation and compression stocking therapy) or, depending on the severity of the deep venous thrombotic symptoms, by catheter directed thrombolysis, followed by first rib resection if thrombolysis was successful. Depending on residual venous lesions, subsequent endovascular treatment (percutaneous transluminal angioplasty with eventual additional stenting) or venous reconstruction was performed after the first rib resection.

ATOS was only treated surgically in symptomatic patients. This could be disabling claudication not responding to supervised exercise therapy of the arm or critical upper extremity ischemia resulting from subclavian artery obstruction or by peripheral embolization caused by a subclavian artery aneurysm. If needed, catheter-directed thrombolysis was performed first, followed by first rib resection, and in case of residual vascular lesions (stenosis or aneurysm), a reconstruction or endovascular treatment was performed subsequently.

The standard surgical approach in both institutions was a first rib resection through a transaxillary approach, as described by Roos et al. [5]. A wound suction drain was usually sufficient to allow complete expansion of the lung if pleural defects occurred during dissection. A chest tube was inserted in cases of persistent pneumothorax. Postoperative pain was managed using a preoperatively given scalene nerve block and intravenous opioids, and early mobilization and physiotherapy were prescribed. In some cases of VTOS, an infraclavicular approach was preferred for better exposure of the subclavian vein [6]. For ATOS and occasionally for cervical ribs, a supraclavicular approach was preferred [6]. Patients were referred to a physiotherapist postoperatively and received instructions to limit abduction to 90° for the first two weeks. After two weeks, no limitations and active mobilization was prescribed. A routine follow-up visit was planned for all patients six weeks postoperatively. 

### 2.3. Long-Term Outcome

For the assessment of the functional and surgical outcomes, all patients were contacted by telephone and were, after agreement for participation was obtained, asked to complete a validated questionnaire. The 11-item version of the Disabilities of the Arm, Shoulder and Hand (QuickDASH) questionnaire was used to assess the subjective disability of arm and shoulder function [7,8,9]. The QuickDASH questionnaire is validated and frequently used in functional studies after upper extremity operations [7,8,9]. A higher score (maximum 100) implies a higher subjective disability of arm and shoulder function. A score of 0 represents optimal function. The questionnaire has two optional modules assessing work and sports, which were only used with patients who worked or were engaged in any sports activities. The questionnaire is available on http://www.dash.iwh.on.ca/about-quickdash.

### 2.4. Statistical Analysis

All patient information was put into a database created in SPSS 23.0 software (IBM, Armonk, NY, USA) for statistical analysis. Continuous variables are expressed as medians with ranges.

## 3. Results

### 3.1. Patient Characteristics

Between January 2005 and December 2016, 56 patients with TOS underwent a first rib resection and 18 also underwent a cervical rib resection. Six patients were treated bilaterally; therefore, the study included a total of 62 surgical procedures. All patients were contacted and asked to complete and return the QuickDASH questionnaire. Follow-up data were available at 30 days for all patients, and all attended the first postoperative visit at four to six weeks. Of the 56 patients, 41 (73%) returned a completely filled-out questionnaire. Reasons for nonresponders included could not be reached by telephone or mail (*n* = 6), refused to complete the questionnaire (*n* = 3), and death from a cause unrelated to the operation or TOS (*n* = 2). Four patients who initially agreed to participate in the study never returned their questionnaires despite several reminders. The interval between the operation and completing the QuickDASH questionnaire was at least one year (range, 1–11 years). Characteristics of the patients who did and did not respond are summarized in Table 1.

### 3.2. Surgical Procedure and Complications

Transaxillary first rib resections were performed in 51 of the 62 surgical procedures. A supraclavicular approach was performed in nine cases to remove cervical ribs (*n* = 6) or reconstruct the subclavian artery (ATOS; *n* = 3). Two patients underwent an infraclavicular approach and subclavian vein reconstruction because of VTOS. In the remaining ATOS (*n* = 10) and VTOS (*n* = 7) patients, an additional percutaneous transluminal angioplasty with stent placement was performed after resection of the first rib. 

Pleural damage during the operation in five patients resulted in a pneumothorax that required chest tube drainage. One patient underwent a reoperation because of postoperative bleeding. No wound infections or other infectious complications occurred, and no transient or permanent motor nerve injury was observed. The 30-day mortality rate was 0.

### 3.3. Functional Outcome and QuickDASH

At long-term follow-up (i.e., ≥1 year), 27 patients (54%) reported complete relief of symptoms. The remaining patients reported minor remaining paresthesia in the ipsilateral arm (*n* = 2), some persisting pain in the ipsilateral arm or shoulder (*n* = 14), or a recurrence of the preoperative complaints (*n* = 8). After 56 of the 62 interventions (90%), patients reported an improvement of their symptoms during the last visit at the outpatient clinic.

The median QuickDASH score for the 41 responders was 22 (range, 0–86). The median score was 18 (range, 0–63) for the work module (*n* = 28) and was 23 (range, 0–100) for the sports module (*n* = 27).

## 4. Discussion

The present study evaluated the long-term functional outcome of surgically treated patients with TOS. A complete resolution of symptoms was reported by 54% of the patients, and clinical improvement was obtained in 90% of patients. Median scores at a follow-up of at least one year postoperatively were 22 for the QuickDASH, 18 for the optional work module, and 22 for the optional sports module. These scores were all slightly higher compared with the values found in the general population in the United States, where these score were 10, 9, and 10, respectively [10]. Apparently, TOS patients continue to experience more functional impairment compared with the general population, despite surgical treatment. Although most patients reported an improvement of symptoms, only 39% had a mean QuickDASH score of 10 or lower. This suggests that even though preoperative symptoms improve, some functional impairment can remain and might be partly caused by the surgical procedure. However, the data of the present study are not sufficient to prove this hypothesis.

Several other studies have reported DASH scores after surgical treatment for TOS ranging from 3.5 to 36 [11,12,13,14,15,16]. Functional outcome as reported by DASH scores is better in athletes [11] and in vascular forms of TOS [14,16]. The explanation for the differences in the outcome between vascular forms of TOS and NTOS is that a substantial number of patients with VTOS or ATOS also undergo revascularization procedures. There is also some evidence indicating that early treatment (≤3 months) might result in better functional outcome than delayed (>6 months) treatment [12,13]. A hypothesis to explain this benefit of early treatment is the prevention of further nerve degeneration and muscle wasting caused by the compression at the thoracic outlet [13]. A meta-analysis by Peek et al. [4] found a mean improvement in the DASH score of 28.3 points after surgical treatment for TOS. Furthermore, an overall clinical success of ≥90% was found for vascular forms of TOS and was 60–80% for the NTOS patients [4].

The diagnosis of NTOS especially remains a challenge for many physicians and might be an important explanation for the relatively disappointing outcomes of surgical treatment of NTOS compared with ATOS and VTOS. Although there are several diagnostic provocation tests, such as the elevation arm stress test and the Adson test, or a trial scalene block, these tests depend on (subjective) patient-reported symptoms. More objective parameters for the diagnosis of NTOS are required. MRI or CT angiography might be used to identify evidence for compression at the thoracic outlet. 

Patient selection could also be improved by obtaining disease-specific validated questionnaires that use discriminating signs and symptoms. Balderman et al. [17] recently described clinical diagnostic criteria that can help to diagnose NTOS. In a cohort of 183 patients referred with NTOS, the most frequently positive pretreatment criteria were neck or upper extremity pain (99%), upper extremity or hand paresthesia (94%), symptom exacerbation by arm elevation (97%), localized supraclavicular or subcoracoid tenderness to palpation (96%), and a positive three-minute elevated arm stress test (94%). Further research is needed to confirm these findings and to correlate these pretreatment criteria with clinical outcome [17].

Another future diagnostic approach could include sensitive nerve conduction studies or imaging studies of the thoracic outlet in different provocative positions of the extremities.

There are several limitations that influence the conclusions that can be drawn from this study. The retrospective nature implies a risk of selection bias, although all consecutively operated-on patients were included. The sample size of 62 patients is reasonable compared with previous studies, although this sample was acquired over a period of approximately 11 years (2005–2016). As a result of the interval between surgery and patients completing the questionnaires, the influence of other factors or events on functional performance cannot be ruled out. Unfortunately, our study did not include data on conservatively managed patients, and therefore, a comparison between conservative and surgical treatment could not be made. Because of the retrospective design, we had no data on baseline QuickDASH scores and could not compare preoperative and postoperative functional outcomes. Finally, the heterogeneity within the study cohort (types of TOS, surgical approach) and the sample size precluded reliable regression analysis to identify factors predictive for a functional outcome.

## 5. Conclusions

In conclusion, long-term functional outcome of surgical treatment in TOS patients is satisfactory, and surgery is beneficial in most patients. However, the mean QuickDASH scores remain higher compared with the general population, suggesting some functional impairment remains despite clinical improvement of symptoms.

## Figures and Tables

**Table 1:** Patient characteristics.

Characteristics		Responders (*n* = 41)	Non-Responders (*n* = 15)
Male sex		16 (39%)	5 (33%)
Age		43 (17–64)	40 (21–64)
ASA 1/2		41 (100%)	13 (87%)
Smoking		14 (34%)	6 (40%)
Type of TOS	NTOS	26 (63%)	10 (67%)
	VTOS	7 (17%)	0 (0%)
	ATOS	10 (24%)	3 (20%)
	Combined	4 (10%)	2 (13%)
Bilateral TOS		6 (14%)	0 (0%)
Cervical rib		13 (28%)	5 (33%)
Athlete		1 (2%)	0 (0%)

Values are *n* (%) or median (range). Abbreviations: TOS—thoracic outlet syndrome; NTOS—neurogenic thoracic outlet syndrome; VTOS—venous thoracic outlet syndrome; ATOS—arterial thoracic outlet syndrome.
